# Safety Assessment and Evaluation of Probiotic Potential of *Lactobacillus bulgaricus* IDCC 3601 for Human Use

**DOI:** 10.3390/microorganisms12102063

**Published:** 2024-10-15

**Authors:** Minjee Lee, Won-Yeong Bang, Han-Bin Lee, Soo-Yeon Yang, Kyu-Shik Lee, Hae-Ji Kang, Sun-Mee Hong, Jungwoo Yang

**Affiliations:** 1 Ildong Bioscience, Pyeongtaek 17957, Republic of Korea; mjleee@ildong.com (M.L.); yeong0417@ildong.com (W.-Y.B.); gksqls9131@ildong.com (H.-B.L.); tndus4679@ildong.com (S.-Y.Y.); 2 Department of Pharmacology, College of Medicine, Dongguk University, Gyeongju 38066, Republic of Korea; there1@dongguk.ac.kr; 3 Department of Microbiology, College of Medicine, Dongguk University, Gyeongju 38066, Republic of Korea; haedi1202@dongguk.ac.kr; 4 Department of Technology Development, Marine Industry Research Institute for East Sea Rim, Uljin 36315, Republic of Korea

**Keywords:** lactic acid bacteria, probiotic, *Lactobacillus bulgaricus*, homemade yogurt, safety evaluation

## Abstract

Lactic acid bacteria (LAB) are probiotic microorganisms widely used for their health benefits in the food industry. However, recent concerns regarding their safety have highlighted the need for comprehensive safety assessments. In this study, we aimed to evaluate the safety of *L. bulgaricus* IDCC 3601, isolated from homemade plain yogurt, via genomic, phenotypic, and toxicity-based analyses. *L. bulgaricus* IDCC 3601 possessed a single circular chromosome of 1,865,001 bp, with a GC content of 49.72%, and 1910 predicted coding sequences. No virulence or antibiotic resistance genes were detected. Although *L. bulgaricus* IDCC 3601 exhibited antibiotic resistance to gentamicin and kanamycin, this resistance is an intrinsic feature of this species. *L. bulgaricus* IDCC 3601 did not produce biogenic amines and did not exhibit hemolytic activity. Phenotypic analysis of enzyme activity and carbohydrate fermentation profiles revealed the metabolic features of *L. bulgaricus* IDCC 3601. Moreover, no deaths or abnormalities were observed in single-dose oral toxicity tests, suggesting that *L. bulgaricus* IDCC 3601 has no adverse effect on human health. Finally, *L. bulgaricus* IDCC 3601 inhibited the growth of potential carbapenem-resistant *Enterobacteriaceae*. Therefore, our results suggest that *L. bulgaricus* IDCC 3601 is a safe probiotic strain for human consumption.

## 1. Introduction

Lactic acid bacteria (LAB) are Gram-positive and facultative anaerobic bacteria, and produce lactic acids as the main products of carbohydrate fermentation [1]. Several genera of bacteria, including *Lactobacillus*, *Leuconostoc*, *Pediococcus*, and *Streptococcus*, are recognized as LAB [2]. LAB have been used in the food industry to produce yogurt, cheese, pickles, beer, and other fermented foods for a long time [3]. LAB not only impact on the flavor of food and its acidity as a preservative agent but also exhibit many health benefits, including the maintenance of mucosal integrity, antipathogenicity, lactose intolerance and food allergy treatment, the prevention of diarrhea, and the suppression of inflammation [4,5,6]. Some genera of LAB, such as *Lacticaseibacillus*, *Lactiplantibacillus*, and *Lactobacillus*, exhibit probiotic characteristics with following general criteria as beneficial microorganisms [7]. First, they must be able to survive and colonize the gastrointestinal tract by resisting the acidic gastric environment [7]. Second, they can modulate or boost host immune response [8]. Third, they must be non-toxic and non-pathogenic to humans [9].

However, few cases associated with the risks of probiotics have been reported. These are mostly manifested in young children and very low birth weight infants; seriously ill adults and infants in intensive care units; and postoperative, hospitalized, or immunocompromised patients, in part due to bacteremia and fungemia [10]. Therefore, safety evaluations are necessary for the safe consumption of probiotic bacteria, and the safety has been scientifically assessed based on the Food and Agriculture Organization of the United Nations (FAO)/World Health Organization (WHO) and European Food Safety Authority (EFSA) guidelines. In general, the FAO/WHO and EFSA guidelines contain antibiotic resistance, hemolytic activity, toxigenicity, and pathogenicity of probiotics [11,12]. Importantly, strain-specific safety assessment is essential for the use of human consumption, since each strain has its own characteristics due to genetic (or phenotypic) difference [13,14]. In this regard, the FAO/WHO suggested to analyze whole genome sequence as a promising approach to identify bacterial strain as well as to identify the risks for virulence factors and the horizontal transferability of antibiotic resistance genes [15,16].

*L. bulgaricus*, first isolated by Bulgarian doctor, Stamen Grigorov in 1905, is a homofermentative organism that produces lactic acids more than 85% of end products and is found naturally in the gastrointestinal tracts of mammals [17,18]. Since Yllia Metchnikoff reported the health benefits of *Lactobacillus* strains in yogurt, *L. bulgaricus* has been wildly studied for yogurt and food supplements [18,19]. For example, recent studies showed that *L. bulgaricus* exerts various beneficial effects, including anti-inflammatory, colitis-associated cancer-inhibiting, and wound-healing effects [20,21,22]. In this study, we aimed to evaluate the safety of *L. bulgaricus* IDCC 3601 isolated from homemade plain yogurt based on the FAO/WHO (2002) and EFSA guidelines (2018) [12,23]. Initially, the whole-genome sequence of *L. bulgaricus* IDCC 3601 was analyzed to identify the virulence and antibiotic resistance genes. In addition, antibiotic susceptibility, biogenic amine (BA) production, hemolytic activity, enzymatic activity, carbohydrate utilization, and antibacterial activity were evaluated to determine the safety of *L. bulgaricus* IDCC 3601. For in vivo safety assessment, acute oral toxicity was assessed using a rat model. Thus, this study suggested that *L. bulgaricus* IDCC 3601 can be used as a food supplement for human consumption, based on our systematic analysis. 

## 2. Materials and Methods

### 2.1. Bacterial Culture Conditions

The *L. bulgaricus* IDCC 3601 (ATCC BAA-2844^TM^) isolated from homemade plain yogurt was anaerobically grown in De Man Rogosa and Sharpe medium (MRS; BD Difco, Franklin Lakes, NJ, USA) at 37 °C. The bacterial cultures of *L. bulgaricus* IDCC 3601 were used for analyses of minimum inhibitory concentration of antibiotics, hemolytic activity, carbohydrate utilization, and extracellular enzyme activity, while the freeze-dried powder of *L. bulgaricus* IDCC 3601 was used for an acute oral toxicity study. To analyze biogenic amine and antibacterial activity, the cell-free supernatant of *L. bulgaricus* IDCC 3601 was used, after centrifugation (4146× *g* for 8 min) of the cell cultures and filtration with a 0.22 μm pore size membrane of cell cultures (Merck Millipore, Burlington, MA, USA).

### 2.2. Whole-Genome Sequencing of L. bulgaricus IDCC 3601

Whole-genome sequencing was performed using the PacBio RS II instrument (Pacific Biosciences of California Inc., Menlo Park, CA, USA) on an Illumina platform (Illumina Inc., San Diego, CA, USA). Average nucleotide identity (ANI) was calculated using an ANI calculator (Kostas Lab, Atlanta, GA, USA). The assembled sequences were compared to the reference antibiotic resistance sequences from the ResFinder database (http://genepi.food.dtu.dk/resfinder) using ResFinder 4.1 software. The search parameters for analysis were as follows: sequence identity > 80% and coverage > 60%. Additionally, virulence genes were searched using the BLASTn algorithm and virulence factor database (http://www.mgc.ac.cn/VFs/). The thresholds for identification were as follows: identity > 80%, coverage > 70%, and *E*-value < 1 × 10^−5^.

### 2.3. Determination of the Minimum Inhibitory Concentration (MIC)

The susceptibility of *L. bulgaricus* IDCC 3601 to ampicillin, vancomycin, gentamicin, kanamycin, streptomycin, erythromycin, clindamycin, tetracycline, and chloramphenicol (Sigma-Aldrich, St. Louis, MO, USA) was assessed. The test was conducted based on the Clinical and Laboratory Standards Institute (CLSI) guidelines [24]. Briefly, a single colony of *L. bulgaricus* IDCC 3601 was inoculated in MRS broth and incubated overnight. The overnight culture was transferred into fresh MRS broth and incubated for 4–6 h. Finally, the cultured cells (5 × 10^5^ CFU/mL) were adjusted using the McFarland standard (BIOMÉRIUX, Marcy-l’Etoile, France). The cultured cells and serially two-fold diluted antibiotics (0.125–1024 µg/mL) were mixed in a 96-well microplate and the plate was anaerobically incubated at 37 °C for 18–20 h. Then, optical density was measured using a microplate reader (Epoch2; BioTek, Winooski, VT, USA). The MIC was determined at the lowest antibiotic concentration that completely inhibited cell growth.

### 2.4. Production of Biogenic Amines (BAs)

To extract BAs, 0.5 mL of cell-free supernatant was mixed with the equivalent of 0.1 M HCl. For derivatization, 1 mL of extracted BAs was incubated at 70 °C for 10 min, followed by the addition of 200 μL of saturated NaHCO_3_, 20 μL of 2 M NaOH, and 0.5 mL of dansyl chloride (10 mg/mL in acetone). Derivatized BAs were mixed with 200 μL of _L_-proline solution (100 mg/mL in H_2_O) and incubated in the dark for 15 min; acetonitrile was added to obtain a final volume of 5 mL. Analysis was performed using a high-performance liquid chromatography system (Agilent 1260; Agilent Technologies, Santa Clara, CA, USA) equipped with a C18 column (YMC-Triart, 4.6 mm × 250 mm; YMC, Kyoto, Japan). An acetonitrile solution (67:33 H_2_O, *v*/*v*) was used as the mobile phase at a constant flow rate of 0.8 mL/min, and a UV detector (G7115A, Agilent Technologies) at 254 nm was used to detect BAs. Quantification of BAs, including tyramine, histamine, putrescine, 2-phenethylamine, cadaverine, and tryptamine, was performed by plotting calibration curves for each BA. 

### 2.5. Hemolytic Activity

The *β*-hemolysis activity of *L. bulgaricus* IDCC 3601 was assessed with the controls of *Staphylococcus aureus* subsp. *aureus* ATCC 25923 (positive) and *L. reuteri* IDCC 3701 (negative). Briefly, single colonies of *L. bulgaricus* IDCC 3601, *S. aureus* ATCC 25923, and *L. reuteri* IDCC 3701 were streaked on a sheep blood agar plate and the plate was incubated at 37 °C for 16 h. Then, hemolysis activity was determined by examining the clear zones around colonies.

### 2.6. Carbohydrates Utilization and Extracellular Enzyme Activities

The carbohydrate utilization by *L. bulgaricus* IDCC 3601 was determined using the API 50 CHL Kit (bioMérieux, Marcy l’Etoile, France) with 49 different carbohydrates. Briefly, cultures of *L. bulgaricus* IDCC 3601 were centrifuged at 4146× *g* for 8 min, and cell pellets (6 × 10^8^ CFU/mL) were suspended in the API 50 CHL medium. The suspended cells were inoculated into a well-type plate provided by the manufacturer, and the plate was incubated at 37 °C for 24 h. The color changes were observed.

Next, extracellular enzyme activities of *L. bulgaricus* IDCC 3601 was determined using the API ZYM kit (bioMérieux) with 19 different substrates. Briefly, cultures of *L. bulgaricus* IDCC 3601 were centrifuged at 4146× *g* for 8 min, and the cell pellet was resuspended in a sterile saline solution to adjust the final cell concentration to 1 × 10^9^ CFU/mL. The suspended cells were inoculated into a well-type plate provided by the manufacturer, and the plate was incubated at 37 °C for 4 h. The ZYM A and ZYM B solutions were sequentially added to the wells, and the color changes were observed after 5 min at room temperature.

### 2.7. Acute Oral Toxicity Study of L. bulgaricus IDCC 3601

The acute oral toxicity of *L. bulgaricus* IDCC 3601 was assessed at the Korea Testing & Research Institute (Hwasun-gun, Jeollanam-do, Korea) according to the Organization for Economic Cooperation and Development guidelines for chemical testing (No. TGK-2023-000259, Figure S1). Twelve female Sprague–Dawley rats (214.5 ± 13.7 g), aged 9–10 weeks, were randomly divided into four groups (three rats per group). An acute dose of *L. bulgaricus* IDCC 3601 was orally administered at 300 mg/kg body weight (4.5 × 10^9^ CFU/kg body weight, 1st and 2nd groups) and 2000 mg/kg body weight (3.0 × 10^10^ CFU/kg body weight, 3rd and 4th groups). Then, mortality, clinical signs, body weight, and necropsy findings were recorded for 14 days. A necropsy was performed to assess physical and clinical damage (e.g., inflammation, tumor, and sepsis by infection exposure) through visual inspection of each organ, following an anesthesia using isoflurane.

### 2.8. Antibacterial Activity against Enterobacteriaceae

The antibacterial activity of *L. bulgaricus* IDCC 3601 was assessed as previously described with minor modifications [25]. Briefly, the cell-free supernatants of *L. bulgaricus* IDCC 3601 were filtered using a 0.22 μm pore size membrane (Merck Millipore). Three bacterial species of *Enterobacteriaceae*, *Klebsiella pneumoniae* subsp. *pneumoniae* KCTC 12385, *Enterobacter cloacae* subsp. *cloacae* KCTC 2631, and *Escherichia coli* KCTC 2441, were cultured in Nutrient medium (BD Difco) at 37 °C for 24–48 h. When the cell density was at 1.5 × 10^8^ CFU/mL using the McFarland standard (bioMérieux), 100 μL of the supernatant from *L. bulgaricus* IDCC 3601 was added to a 96-well plate, along with an equal volume of each pathogen suspension. The plates were incubated at 37 °C for 48 h, and then the optical density (OD_600_) was measured using a microplate reader (BioTek). The antibacterial activity was determined by calculating the ratio of the OD_600_ at 48 h to the OD_600_ at 0 h.

### 2.9. Statistical Analysis

The results are expressed as the mean ± standard deviation from biological triplicates. Statistical significance between groups was determined by an unpaired two-tailed *t*-test using GraphPad Prism 10 (GraphPad Software Inc., La Jolla, CA, USA). A significant difference was defined as *p* < 0.05.

## 3. Results and Discussion

### 3.1. Genomic Analysis of L. bulgaricus IDCC 3601

Bacterial genome analysis provides a comprehensive understanding of the metabolic pathway, microbial physiology, and potential application along with species identity [13,26]. Here, the genome of *L. bulgaricus* IDCC 3601 was found to possess a single circular DNA chromosome of 1,865,001 bp with 49.72% of GC content and 1910 predicted coding DNA sequences (Table S1). As shown in Figure S2 and Table S2, the genes were functionally annotated using the evolutionary gene genealogy non-supervised orthologous groups (EggNOG) mapper v2 (http://eggnog-mapper.embl.de, accessed on 8 October 2024). As expected, a significant portion of the total genes was involved in essential biological functions. More specifically, 772 (40.44%) were assigned to five major EggNOG functional categories: replication, recombination, and repair (L, 11.63%); carbohydrate transport and metabolism (G, 4.56%); amino acid transport and metabolism (E, 8.96%); transcription (K, 7.23%); translation, ribosomal structure, and biogenesis (J, 8.07%). 

Identification of antibiotic resistance gene(s) (e.g., β-lactamase and efflux pump) in a dietary microbe is important as these factors are often encoded by genes located on conjugative plasmids, posing a potential risk of gene transfer [27]. Previously, it was demonstrated that few *Lactobacillus* species carry antibiotic resistance genes (e.g., *tetL and tetW*), which are responsible for resistance to several antibiotics (e.g., tetracycline) [28,29]. Here, the genome of *L. bulgaricus* IDCC 3601 does not harbor the gene sequences associated with virulence (e.g., toxin) and antibiotic resistance. 

### 3.2. Antibiotic Susceptibility

Next, the antibiotic resistance of *L. bulgaricus* IDCC 3601 was evaluated based on MIC values (μg/mL), exceeding the recommended cut-off values defined by EFSA [12]. The MIC values of *L. bulgaricus* IDCC 3601 against nine antibiotics were slightly lower than or equal to the cut-off values defined by EFSA (Table 1). Antibiotic resistance profiles indicated that *L. bulgaricus* IDCC 3601 was susceptible to all the tested antibiotics, except gentamicin and kanamycin. Most *Lactobacillus* species are inherently resistant to aminoglycosides, including gentamicin and kanamycin [30]. Intrinsic antibiotic resistance is not transferrable to commensal microbes, because it is attributed to the specific membrane characteristics of the bacteria, and the absence of cytochrome-mediated electron transport that facilitates the absorption of antibiotics [31,32]. Thus, *L. bulgaricus* IDCC 3601 can be considered a safe strain with respect to antibiotic resistance.

### 3.3. Determination of Biogenic Amines (BAs)

BAs are organic, basic, and nitrogenous compounds found in various fermented foods that can induce various adverse effects, including headaches, hypertension, and allergic reactions, when they are overproduced [33]. Several studies, including those by EFSA, have highlighted the importance of using probiotic strains that do not produce BAs [34]. In this study, BAs analysis, such as tyramine, histamine, putrescine, 2-phenethylamine, cadaverine, and tryptamine revealed that *L. bulgaricus* IDCC 3601 did not produce detectable levels of these BAs (Table S3). The absence of BAs production by *L. bulgaricus* IDCC 3601 is consistent with a previous report on the lack of production of BAs by *L. bulgaricus* strains [35]. 

### 3.4. Hemolytic Property

*β*-Hemolysis is one of the pathogeneses (i.e., invasion) inducing the destruction of host red blood cells and the leakage of hemoglobin, and spreading pathogens throughout the body. An absence of hemolytic activity is a positive attribute reflecting the non-pathogenic nature of probiotic strains [36]. As shown in Figure S3, *L. bulgaricus* IDCC 3601 exhibited no *β*-hemolytic activity, as no clear zones were formed around the colonies on sheep blood agar plates. In contrast, *S. aureus*, the positive control, showed hemolytic activity by forming a clear zone on the same plates. Many strains of *Lactobacillus*, including *L. bulgaricus*, typically do not possess hemolysin, exhibiting hemolytic activity [37]. 

### 3.5. Carbohydrate Utilization and Extracellular Enzymatic Activities

Lactic acid bacteria utilize carbohydrates via either homofermentative or heterofermentative metabolism. *L. bulgaricus*, known as a homofermentative bacterium, produces lactic acids as the primary by-products [38]. The carbohydrate utilization phenotype of *L. bulgaricus* IDCC 3601 was evaluated using the API 50 CHL kit. *L. bulgaricus* IDCC 3601 can utilize hexoses such as d-glucose, d-fructose, d-mannose, while it cannot utilize pentoses such as xylose, ribose, and arabinose (Table 2). Among the disaccharides, it can utilize only lactose due to the presence of *β*-galactosidase.

The extracellular enzyme activities of *L. buglaricus* IDCC 3601 was further evaluated using the API ZYM kit. In results, *L. bulgaricus* IDCC 3601 exhibited enzymatic activities related to lipid (esterase), aminopeptidase (leucine arylamidase, valine arylamidase, and cystine arylamidase), vitamin (acid phosphatase), phosphate (naphthol-as-bi-phosphohydrolase), and carbohydrate (*β*-galactosidase) metabolism (Table 3). Aminopeptidases, such as leucine arylamidase, valine arylamidase, and cystine arylamidase, reduce the bitterness and improve the flavor of cheese [39,40]. *β*-galactosidase hydrolyzes lactose into glucose and galactose, which alleviates lactose intolerance. In a previous study, two *L. bulgaricus* strains were found to possess the *lacZ* operon, showing *β*-galactosidase activity [41]. Additionally, *β*-glucuronidase catalyzes the hydrolysis of *β*-glucuronosyl-O-links, releasing potential carcinogenic substances into the colon [42,43]. Importantly, *L. bulgaricus* IDCC 3601 exhibited no *β*-glucuronidase activity.

### 3.6. Acute Oral Toxicity Test

**Figure 1:**
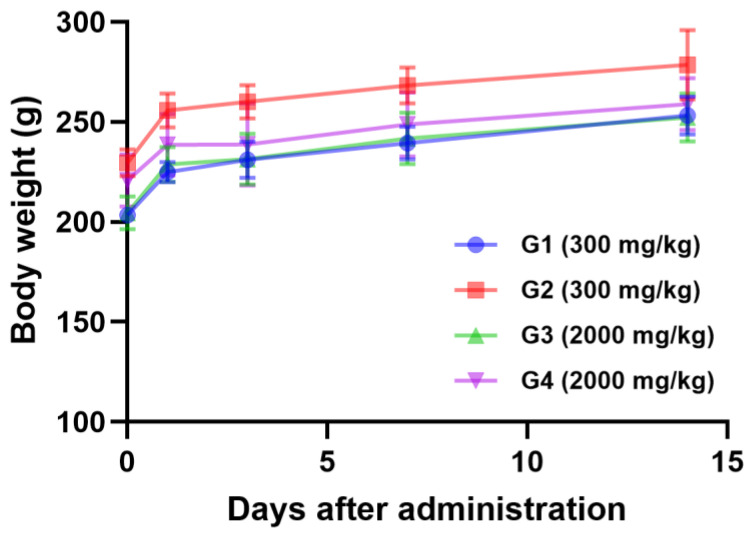
Body weight changes in a single-dose acute oral toxicity study of *Lactobacillus bulgaricus* IDCC 3601. G1, 300 mg/kg, 9 weeks; G2, 300 mg/kg, 10 weeks; G3, 2000 mg/kg, 9 weeks; G4, 2000 mg/kg, 10 weeks.

The acute oral toxicity of *L. bulgaricus* IDCC 3601 was investigated using a single-dose acute oral toxicity test. As shown in Figure 1, although there was a slight body weight loss of 0.08–2.19% in the G4 group at 1–3 days, overall body weight increased without any visible adverse events. The weight loss in G4 group was considered incidental and transient in some animals based on the clinical signs, degree of body weight reduction, and necropsy findings. Moreover, no mortality or clinical signs were observed in all groups following the oral administration of *L. bulgaricus* IDCC 3601. The observed changes were not affected by the administration of *L. bulgaricus* IDCC 3601. At necropsy, no abnormal changes were observed in any of the groups. 

### 3.7. Antibacterial Activity

**Figure 2:**
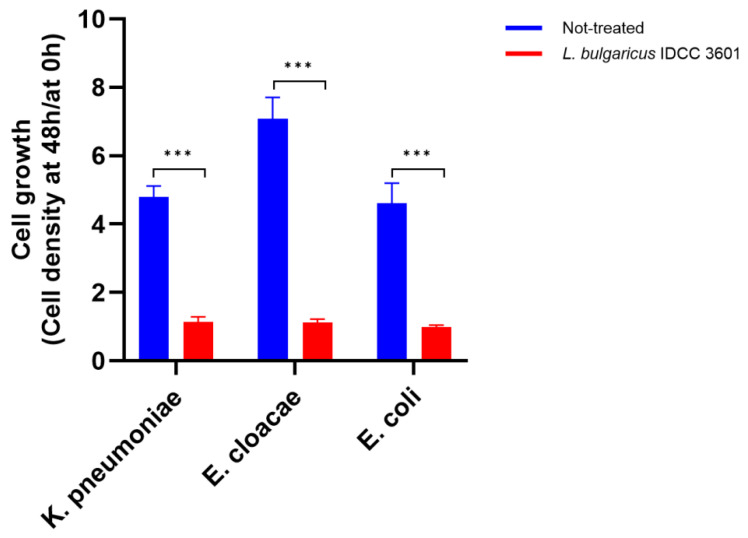
The antibacterial activity of *Lactobacillus bulgaricus* IDCC 3601 against *Klebsiella pneumoniae* subsp. *pneumoniae* KCTC 12385, *Enterobacter cloacae* subsp. *cloacae* KCTC 2631, and *Escherichia coli* KCTC 2441. The results are expressed as means ± standard deviations of three independent experiments. Statistical significance was determined by an unpaired two-tailed *t*–test. *** *p* < 0.001.

For a probiotic bacterium, several prerequisites exist including survival during gastrointestinal transit, intestinal settlement, and antibacterial activity [14]. Previously, we showed that (1) *L. bulgaricus* IDCC 3601 can survive 81% and 93% under acid and bile conditions, respectively, (2) it can adhere at 47% onto Caco-2 cells, and (3) it can inhibit *Salmonella* Typhimurium [44,45]. Here, we tested whether *L. bulgaricus* IDCC 3601 could inhibit the growth of *Enterobacteriaceae* such as *K. pneumoniae* KCTC 12385, *E. cloacae* KCTC 2631, and *E. coli* KCTC 2441. In results, the growth rates of *K. pneumoniae* KCTC 12385, *E. cloacae* KCTC 2631, and *E. coli* KCTC 2441 significantly decreased by 79%, 76%, and 84%, respectively, compared with the control (Figure 2). 

*Enterobacteriaceae* are mostly opportunistic pathogens and typically exhibit multidrug resistance. Recent prevalence studies highlighted that patients with compromised immune system are at an increased risk of infections by multidrug-resistant organisms, such as *Enterobacteriaceae* due to antibiotic overuse [46]. More seriously, *Enterobacteriaceae* can acquire resistance to carbapenems, which are a class of highly effective β-lactam antibiotics used to treat severe or high-risk bacterial infections. Thus, carbapenem-resistant *Enterobacteriaceae* (CRE) are recognized as life-threatening bacteria, involved in the pathogenesis of pneumoniae and sepsis [47,48]. Recent studies have increasingly focused on probiotics to utilize their properties, such as the production of antimicrobial compounds and reduction in intestinal pH [49]. *L. bulgaricus* strains have been reported to exhibit antimicrobial activity against a broad spectrum of pathogens [50], but its effects on CRE strains have not yet been widely documented. This study evaluated the antimicrobial activity of *L. bulgaricus* IDCC 3601 against three *Enterobacteriaceae* species. 

These results are comparable to those of *L. delbrueckii* subsp. *delbrueckii* LDD01 against *K. pneumoniae* [51], and *L. delbrueckii* DSM 20074 against *E. coli*, *K. pneumoniae*, *K. oxytoca*, and *E. cloaca* [52]. In terms of defense mechanisms, polymorphonuclear cells (PMNs) are primarily responsible for innate immunity of invasive pathogenic bacteria in damaged intestinal tissue. Several studies showed synergism between PMNs and some antibiotics (e.g., ciprofloxacin), inhibiting bacterial proliferation and enhancing antibacterial activity [53,54]. In this regard, antibacterial activity of *L. bulgaricus* IDCC 3601 might be prevention effects on pathogenic infection and might be synergisms with PMNs due to its antibacterial substances (e.g., bacteriocin). 

Regarding the antimicrobial activity of probiotics against CRE, there are several in vivo and clinical studies. For example, *Lactobacillus sakei* PMC104 significantly reduced infection-derived severity by carbapenem-resistant *Klebsiella pneumoniae* [55]. Furthermore, a clinical study examined the use of *Lactobacillus acidophilus* and *Bifidobacterium bifidum* to prevent the intestinal colonization of carbapenemase-producing *Enterobacteriaceae* (CPE) in chronic and long-term carriers [56]. Further studies are needed to clarify the inhibitory effects of *L. bulgaricus* IDCC 3601 against CRE infections.

## 4. Conclusions

Based on genomic, phenotypic, and toxicological analyses, *L. bulgaricus* IDCC 3601 was proven to safe for human consumption, in terms of antibiotic resistance, β-hemolytic activity, toxic compound formation, and acute oral toxicity. Based on results showing (1) a survivability of 81% and 93% under acid and bile conditions, respectively, (2) adhesion at 47% onto human intestinal cells, (3) the growth inhibition of *Salmonella* Typhimurium, and (4) the antibacterial activity of potential carbapenemase-resistant *Enterobacteriaceae* (CRE), *L. bulgaricus* IDCC 3601 could be suggested as a probiotic strain suitable for use as a health functional food.

## Figures and Tables

**Table 1:** Minimum inhibitory concentrations (MICs) of various antibiotics for *Lactobacillus bulgaricus* IDCC 3601.

	AMP ^d^	VAN	GEN	KAN	STR	ERY	CLI	TET	CHL
Cut-off value (µg/mL) ^a^	2	2	16	16	16	1	4	4	4
*L. bulgaricus* IDCC 3601	<0.125/S ^b^	0.25/S	32/R ^c^	64–128/R	16/S	<0.125/S	<0.125/S	1/S	2/S

^a^ European Food Safety Authority (EFSA), 2018. *EFSA Journal*, 16(3), 5206. ^b^ S, susceptible. ^c^ R, resistant. ^d^ Abbreviations: AMP, ampicillin; VAN, vancomycin; GEN, gentamicin; KAN, kanamycin; STR, streptomycin; ERY, erythromycin; CLI, clindamycin; TET, tetracycline; CHL, chloramphenicol.

**Table 2:** Carbohydrate utilization activities of *Lactobacillus bulgaricus* IDCC 3601.

No.	Substrate	Result	No.	Substrate	Result	No.	Substrate	Result
1	Glycerol	–	18	Mannitol	–	35	d-Raffinose	–
2	Erythritol	–	19	Sorbitol	–	36	Amidon	–
3	d-Arabinose	–	20	α-Methyl-D-mannoside	–	37	Glycogene	–
4	l-Arabinose	–	21	α-Methyl-D-glucoside	–	38	Xylitol	–
5	Ribose	–	22	N-Acethyl-glucosamine	+^W a^	39	Gentibiose	–
6	d-Xylose	–	23	Amygdaline	–	40	d-Turanose	–
7	l-Xylose	–	24	Arbutine	–	41	d-Lyxose	–
8	Adonitol	–	25	Esculine	+	42	d-Tagatose	–
9	β-Methyl-xylose	–	26	Salicine	–	43	d-Fucose	–
10	Galactose	–	27	Cellobiose	–	44	l-Fucose	–
11	d-Glucose	+	28	Maltose	–	45	d-Arabitol	–
12	d-Fructose	+	29	Lactose	+	46	l-Arabitol	–
13	d-Mannose	+	30	Melibiose	–	47	Gluconate	–
14	l-Sorbose	–	31	Sucrose	–	48	2-Keto-gluconate	–
15	Rhamnose	–	32	Trehalose	–	49	5-Keto-gluconate	–
16	Dulcitol	–	33	Inuline	–			
17	Inositiol	–	34	Melizitose	–			

^a^ W, weak positive.

**Table 3:** Extracellular enzyme activities of *Lactobacillus bulgaricus* IDCC 3601.

No.	Enzyme	Result	No.	Substrate	Result
1	Alkaline phosphatase	–	11	Naphthol-AS-BI-phosphohydrolase	+
2	Esterase	+	12	*α*-galactosidase	–
3	Esterase lipase	–	13	*β*-galactosidase	+
4	Lipase	–	14	*β*-glucuronidase	–
5	Leucine arylamidase	+	15	*α*-glucosidase	–
6	Valine arylamidase	+	16	*β*-glucosidase	–
7	Cystine arylamidase	+	17	N-acetyl-*β*-glucosaminidase	–
8	Trypsin	–	18	*α*-mannosidase	–
9	α-chymotrypsin	–	19	*α*-fucosidase	–
10	Acid phosphatase	+			

## Data Availability

No new data were created or analyzed in this study. Data sharing is not applicable to this article.

## Supplementary Materials

The following supporting information can be downloaded at: https://www.mdpi.com/article/10.3390/microorganisms12102063/s1, Table S1: Genomic features of Lactobacillus bulgaricus IDCC 3601; Table S2. Functional genes of Lactobacillus bulgaricus IDCC 3601; Table S3. Biogenic amines production by Lactobacillus bulgaricus IDCC 3601; Figure S1. The GLP statement and quality assurance statements for acute oral toxicity study of Lactobacillus bulgaricus IDCC 3601; Figure S2. Functional categorization of Lactobacillus bulgaricus IDCC 3601 using the evolutionary gene genealogy non-supervised orthologous groups (EggNOG) database; Figure S3. No hemolytic activity of Lactobacillus bulgaricus IDCC 3601.
