# Supplementary material for: Safety Assessment and Evaluation of Probiotic Potential of Lactobacillus bulgaricus IDCC 3601 for Human Use
Source: Microorganisms. 2024 Oct 15;12(10):2063. doi: 10.3390/microorganisms12102063 (PMC11510087; doi:10.3390/microorganisms12102063)
Supplement: Supplementary file 1 [file microorganisms-12-02063-s001.zip › microorganisms-3231903-supplementary.pdf]

Supplementary Materials

Table S1. Genomic features of *Lactobacillus bulgaricus* IDCC 3601

Item	Result
Identification	*Lactobacillus bulgaricus*
Genome size (bp)	1,865,001
GC content (%)	49.72
CDS	1,910

Table S2. Functional genes of *Lactobacillus bulgaricus* IDCC 3601

Eggnog	Description	Count	Ratio (%)
J	Translation, ribosomal structure and biogenesis	154	8.0671
A	RNA processing and modification	0	0.0000
K	Transcription	138	7.2289
L	Replication, recombination and repair	222	11.6291
B	Chromatin structure and dynamics	0	0.0000
D	Cell cycle control, cell division, chromosome partitioning	27	1.4144
Y	Nuclear structure	0	0.0000
V	Defense mechanisms	66	3.4573
T	Signal transduction mechanisms	50	2.6192
M	Cell wall/membrane/envelope biogenesis	89	4.6621
N	Cell motility	7	0.3667
Z	Cytoskeleton	0	0.0000
W	Extracellular structures	0	0.0000
U	Intracellular trafficking, secretion, and vesicular transport	25	1.3096
O	Posttranslational modification, protein turnover, chaperones	38	1.9906
C	Energy production and conversion	63	3.3002
G	Carbohydrate transport and metabolism	87	4.5574
E	Amino acid transport and metabolism	171	8.9576
F	Nucleotide transport and metabolism	126	6.6003
H	Coenzyme transport and metabolism	49	2.5668
I	Lipid transport and metabolism	50	2.6192
P	Inorganic ion transport and metabolism	86	4.5050
Q	Secondary metabolites biosynthesis, transport and catabolism	8	0.4191
R	General function prediction only	0	0.0000
S	Function unknown	454	23.7821
Total	-	1,910	100

Table S3. Biogenic amines production by *Lactobacillus bulgaricus* IDCC 3601

**Strain**	**Biogenic amine (mM)**					
	**Tyramine**	**Histamine**	**Putrescine**	**2-Phenethylamine**	**Cadaverine**	**Tryptamine**
L. bulgaricus IDCC 3601	N.D.[a]	N.D.	N.D.	N.D.	N.D.	N.D.

[a]N.D., not detected.

GLP Statement

Study title : Acute oral toxicity study (Acute toxic class method) of *Lactobacillus bulgaricus* IDCC 3601 in rat

Study number : TGK-2023-000259

Sponsor

Name : ILDONG BIOSCIENCE CO., LTD

Address : 17, Poseunggongdan-ro, Poseug-eup, Pyeongtaek-si, Gyeonggi-do, Republic of Korea

Representative : Lee Jang-hyey

Person in charge : Kim Yo-hwan

Department / Position : Quality Control Team / Manager

Contact number : Tel. +82-31-646-3131 Fax. +82-70-7500-2592

Test facility

Name : Korea Testing and Research Institute, Hwasun

Address : 12-63, Sandan-gil, Hwasun-eup, Hwasun-gun, Jeollanam-do, KOREA

Test facility management : Lee Seung-young

Contact number : Tel. +82-61-370-7700 Fax. +82-61-370-7777

This study was conducted under the supervision of the study director and is in compliance with the principles of Good Laboratory Practice.

1. Good Laboratory Practice

1.1. OECD "Principles of Good Laboratory Practice, ENV/MC/CHEM (98)17 (as revised in 1997)"

2. Test regulation

2.1. OECD Guideline for Testing of Chemicals, Section 4, TG 423 "Acute Oral Toxicity-Acute Toxic Class Method" (Adopted : 17th December 2001)

This report acknowledges that there was a deviation from the approved test schedule as specified in the test plan. However, it is important to note that this deviation did not have any impact on the test results. Additionally, we have verified that no circumstances arose during the test execution that could have compromised its reliability.

Study director *(Signed in the original report)* (Annex 7)
Cho Jeong-seong, B.S   Date

※ *This report was translated a Korean sentence into English according to sponsor' request by study director. The original data had not been changed.*

Test facility management *(Signed in the original report)* (Annex 7)
Lee Seung-young, M.S   Date

Translator Cho Jeong-seong   2023-09-22
Cho Jeong-seong, B.S   Date

Quality Assurance Statement

Study title : Acute oral toxicity study (Acute toxic class method) of *Lactobacillus bulgaricus* IDCC 3601 in rat

Study number : TGK-2023-000259

Study Phases	Inspections	Reports to study director and management
Study plan audit	2023-07-06	2023-07-06
Animal receipt audit	2023-07-06	2023-07-06
Group assignment (1) audit	2023-07-10	2023-07-10
Preparation of test substance (1) audit	2023-07-10	2023-07-10
Preparation of test substance (2) audit	2023-07-11	2023-07-11
Test substance administration (1) audit	2023-07-11	2023-07-11
Clinical signs (1) audit	2023-07-11	2023-07-11
Necropsy (1) audit	2023-07-25	2023-07-25
Group assignment (2) audit	2023-07-26	2023-07-26
Test substance administration (2) audit	2023-07-27	2023-07-27
Clinical signs (2) audit	2023-07-27	2023-07-27
Necropsy (2) audit	2023-08-18	2023-08-18
Raw data audit	2023-09-07	2023-09-07
Draft final report audit	2023-09-07	2023-09-07
Final report audit	2023-09-08	2023-09-08

Inspections of the routine and repetitive procedures that constitute the study was carried out as a continuous process designed to encompass the major phases at or about the time this study was in progress.

This report has been audited by KTR Quality Assurance Unit, and is considered to be an accurate account of the raw data generated and of the procedures followed.

Inspections were accomplished as noted, and reported to the study director and management immediately following their completion. Based on these inspections and the review of the report, this study was conducted and reported in conformance with the Good Laboratory Practice regulations.

Quality assurance personnel *(Signed in the original report)* (Annex 8)
Kim Yong-woo, A.S.   Date

Figure S1. The GLP statement and quality assurance statements for acute oral toxicity study of *Lactobacillus bulgaricus* IDCC 3601.

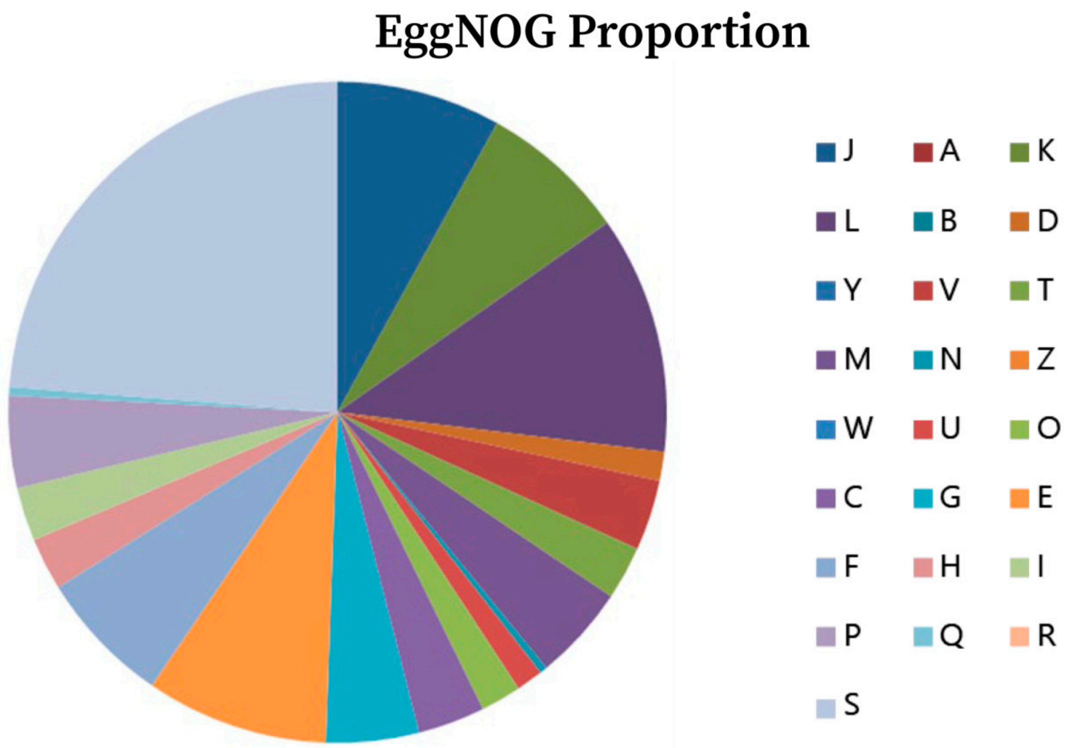

Figure S2. Functional categorization of *Lactobacillus bulgaricus* IDCC 3601 using the evolutionary gene genealogy non-supervised orthologous groups (EggNOG) database. J, Translation, ribosomal structure and biogenesis; A, RNA processing and modification; K, Transcription; L, Replication, recombination, and repair; B, Chromatin structure and dynamics; D, Cell cycle control, cell division, and chromosome partitioning; Y, Nuclear structure; V, Defense mechanisms; T, Signal transduction mechanisms; M, Cell wall/membrane/envelope biogenesis; N, Cell motility; Z, Cytoskeleton; W, Extracellular structures; U, Intracellular trafficking, secretion, and vesicular transport; O, Post-translational modifications, protein turnover, and chaperones; C, Energy production and conversion; G, Carbohydrate transport and metabolism; E, Amino acid transport and metabolism; F, Nucleotide transport and metabolism; H, Coenzyme transport and metabolism; I, Lipid transport and metabolism; P, Inorganic ion transport and metabolism; Q, Secondary metabolite biosynthesis, transport, and catabolism; R, General function prediction only; S, Unknown function

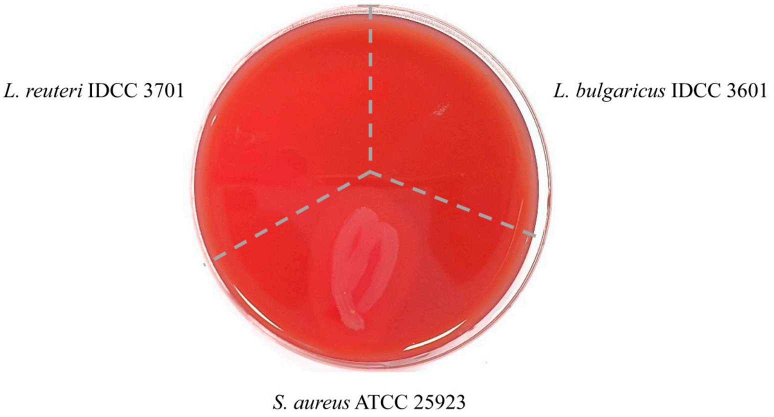

Figure S3. No hemolytic activity of *Lactobacillus bulgaricus* IDCC 3601. *Staphylococcus aureus* subsp. *aureus* ATCC 25923 (positive control) showed β-hemolytic activity, while *Lactobacillus reuteri* IDCC 3701 (negative control) showed γ-hemolytic activity.
